# Preemptive analgesia in dental implant surgery: A systematic review and meta-analysis of randomized controlled trials

**DOI:** 10.4317/medoral.24639

**Published:** 2021-08-19

**Authors:** Gustavo Henrique Mattos-Pereira, Carolina Castro Martins, Rafael Paschoal Esteves-Lima, Rachel Alvarenga-Brant, Luís Otávio Miranda Cota, Fernando Oliveira Costa

**Affiliations:** 1 ORCID: 0000-0003-0176-0741. DDS, MS, PhD candidate. Department of Clinical Dentistry, Pathology and Oral Surgery, Faculty of Dentistry, Federal University of Minas Gerais, Belo Horizonte, Brazil; 2 ORCID: 0000-0001-9072-3226. DDS, MS, PhD, Associate Professor. Department of Pediatric Dentistry, Faculty of Dentistry, Federal University of Minas Gerais, Belo Horizonte, Brazil; 3 ORCID: 0000-0003-4343-3845. DDS, MS, PhD, Adjunct Professor. Department of Clinical Dentistry, Pathology and Oral Surgery, Faculty of Dentistry, Federal University of Minas Gerais, Belo Horizonte, Brazil; 4 ORCID: 0000-0001-9358-2262. DDS, Master student. Department of Clinical Dentistry, Pathology and Oral Surgery, Faculty of Dentistry, Federal University of Minas Gerais, Belo Horizonte, Brazil; 5 ORCID: 0000-0003-1517-5842. DDS, MS, PhD, Associate Professor. Department of Clinical Dentistry, Pathology and Oral Surgery, Faculty of Dentistry, Federal University of Minas Gerais, Belo Horizonte, Brazil; 6 ORCID: 0000-0002-7687-1238. DDS, MS, PhD, Titular Professor. Department of Clinical Dentistry, Pathology and Oral Surgery, Faculty of Dentistry, Federal University of Minas Gerais, Belo Horizonte, Brazil

## Abstract

**Background:**

To assess the effectiveness of preemptive analgesia in dental implant surgery in randomized controlled trials (RCTs).

**Material and Methods:**

The present study was conducted in accordance with the guidelines of the Preferred Reporting Items for Systematic Reviews and Meta-Analyses (PRISMA) statement and registered in PROSPERO database CRD42020168757. A search without restrictions regarding language or date of publication was conducted in six databases and gray literature. A random effect meta-analysis compared the efficacy of preemptive analgesia compared to placebo through pooled OR and 95%CI. The interpretation of results followed the certainty of evidence using the Grading of Recommendations, Assessment, Development and Evaluation (GRADE) approach together with the magnitude of the effect according to GRADE guidelines.

**Results:**

Four studies were included in the review and three were incorporated into the meta-analysis. All studies demonstrated that preemptive analgesia contributed to a significant improvement in the postoperative pain control. However, the overall pooled standard mean difference (SMD) showed that preemptive analgesia had small effects compared to placebo in reducing pain (SMD: -0.45; IC: -0.83; -0.08) with low certainty of the evidence. Our meta-analysis showed that the magnitude of the effect was bigger six to eight hours after the surgery (large effect), compared to the time of one to two hours after the surgery (small effect).

**Conclusions:**

Preemptive analgesia may have a positive effect in reducing pain compared to not using preemptive medication, but the evidence is very uncertain.

** Key words:**Preemptive analgesia, postoperative pain, dental implant surgery, systematic review.

## Introduction

Acute postoperative pain is a normal response to surgical interventions. It is one of the causes of late recovery and post-operative analgesic medication use, often indiscriminate (1).

Among the alternatives for improving postoperative pain control, preemptive analgesia has stood out. Preemptive analgesia consists of administering analgesic medication before tissue injury, that is, before the reception, transmission, modulation, and nociception of the aggressive stimulus, aiming to prevent hyperalgesia and the consequent stimulus that amplifies pain (2). Preemptive analgesia has been used as an effective for pain control method in third molar surgeries (3), also presenting itself as a viable protocol for dental implant surgeries (4).

In implant dentistry, the installation of dental implants comprises a surgical procedure that can generate mild to moderate pain sensations, but which may exceed the normal thresholds in some moments (2). In order to establish an efficient preemptive analgesic effect, it is necessary that an ideal level of antinociceptive medication is administered before the injury and that it remains in the postoperative phase, thus preventing sensitization during the inflammatory phase (5).

There are several groups of analgesic agents used in dentistry, as well as different dosages to be implemented. Therefore, defining which one has the best therapeutic efficacy remains a challenge for dental surgeons (1,3,6).

Therefore, due to the conflicting results in the literature, this systematic review and meta-analysis aims to evaluate the effectiveness of preemptive analgesia in dental implant surgeries.

## Material and Methods

- Protocols and Records

These systematic review and meta-analysis were conducted in accordance with the recommendations of the Preferring Reporting Items for Systematic Reviews and Meta-analyzes (PRISMA) (7). A study protocol was registered with the International Prospective Register of Systematic Reviews PROSPERO, under the number CRD42020168757.

- Eligibility criteria

The clinical question (PICO question) was as follows: "Does preemptive analgesia decrease postoperative pain in patients undergoing dental implant surgery?"

Patient (P): patients undergoing dental implant surgery; Intervention (I): preemptive analgesia using any medication evaluated in clinical trials; Comparison (C): patients who used a placebo as preemptive medication or no treatment; Outcome (O): postoperative pain.

- Inclusion criteria

We included parallel arm randomized placebo-controlled trials (RCTs), with healthy patients above 18 years old, and who had undergone surgery to insert dental implants (single or multiple), who received any type of preemptive medication (analgesic or anti-inflammatories), considering that the medication administration time was not established. No minimum follow-up time of the studies was determined.

The exclusion criteria were non-randomized trials and observational studies, studies without placebo or no treatment as a control group, animal studies, systematic reviews, literature reviews, conference proceedings, editorials, and studies that were not written in the English language.

- Information sources

An electronic search was conducted from the intersection up to January 2020 and updated in December 2020 in the following databases: MedLine through PubMed, Scopus (Elsevier), Web of Science, Latin American and Caribbean Health Sciences Literature (Lilacs) through Virtual Health Library (Bireme), Cochrane Central Register of Controlled Trials (CENTRAL) and Cochrane Database of Systematic Reviews. A manual search was conducted on the included studies and reviews. No restrictions were imposed on the year of publication. The research strategies used in each database are displayed in Table 1.

- Selection of studies

All the retrieved articles were organized in the EndNote Web program.

The selection of the studies took place in two phases and was conducted by two independent reviewers (GHMP and RPEL). First, the reviewers screened titles and abstracts for eligibility. Studies without enough information to make a decision were selected for full-text screening. Full texts were obtained and analyzed for further selection. In the case of disagreement, the reviewers discussed the eligibility criteria until a consensus was reached.

**Table 1:**
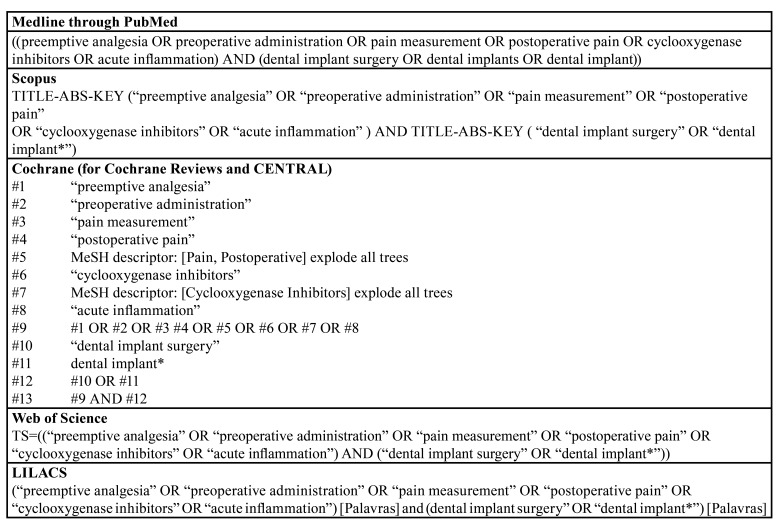
Terms used in the search.

- Data extraction

Two independent reviewers extracted data (GHMP and RPEL). The following data were extracted from each study in an excel spreadsheet previously created: name of the author (s) and year of publication, country, sample size, sex, age, duration of surgery, anesthetic drug used, drop-outs, tested medication, rescue medication, time when preemptive medication was administered, methods for assessing pain and adverse effects.

- Risk of Bias Assessment

The risk of bias was assessed using the Cochrane Bias Risk Tool for Randomized Trials (RoB 2.0) and judged as "low", "some concerns” or “high” (8).

- Outcomes

The primary outcomes were pain from one to two hours after surgery and from six to eight hours after surgery. For each outcome we extracted mean or median scores of pain and respective standard deviation (SD), standard error (SE) or 95% CI, when reported by authors. We used the software Review Manager version 5.4 for meta-analysis for these two primary outcomes. The mean and standard deviation were used to calculate the pooled standard mean difference (SMD) and its 95%CI. We used random effect model considering uncertainty in I2 test for heterogeneity when few studies are used. Moreover, the diversity may occur in meta-analysis with ineviTable heterogeneity (8). We subgrouped each outcome for the type of drug comparing to placebo or no medication as the comparison group.

- Summary of results and the certainty of the evidence

The certainty of the evidence was assessed through Grading of Recommendations, Assessment, Development and Evaluation tool (GRADE) (9).

RCTs start with high certainty of evidence, and it can be rated down due to problems as risk of bias, inconsistency, indirectness, imprecision, and publication bias. Each outcome was tabulated in a Summary of Finding (SoF) Table using GRADEpro. For the magnitude of the effect, we used the Cohen’s effect sizes for SMD: from -0.2 to 0.2 representing a trivial or no effect; -0.5 to -0.2 or 0.2 to 0.5 representing a small effect; -0.8 to -0.5 or 0.5 to 0.8 representing a moderate effect; and < -0.8 or > 0.8 representing a large effect (10). The interpretation of the results was based on an integrated approach of the magnitude of the effect based on Cohen’s effect size and the certainty of the evidence (11).

## Results

- Selection of studies

**Figure 1:**
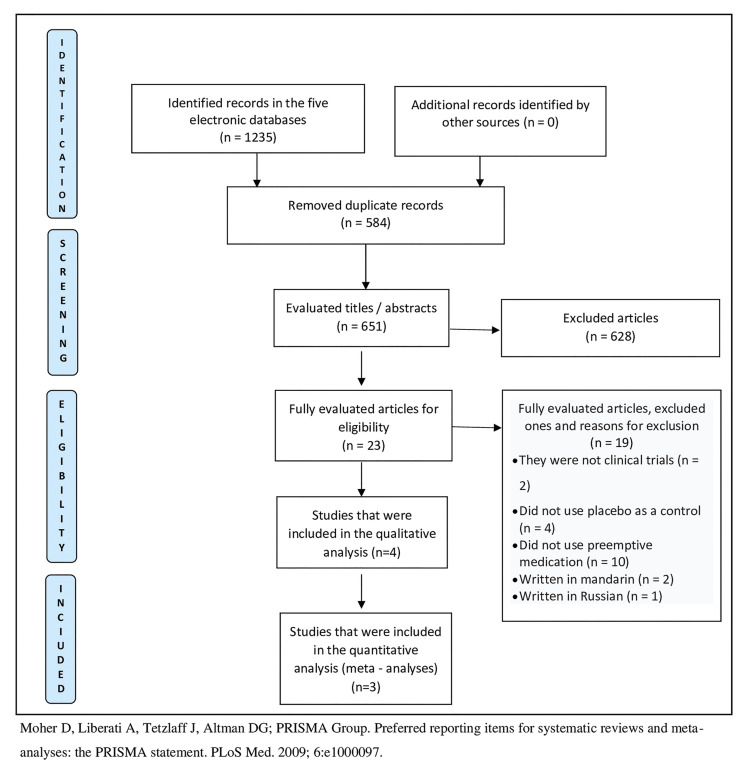
Preferred Reporting Items for Systematic Reviews and Meta-Analyses (PRISMA) flowchart of study screening selection.

One thousand two hundred and thirty five studies were retrieved in the electronic search. After analyzing titles and abstracts, twenty three studies were selected for evaluation through full reading. The main reasons for exclusion after full texts evaluation were studies that: did not use preemptive medication or placebo as a control group, studies that were not clinical trials, and those not written in English. Four studies were included (4,12-14) in the review and three in the meta-analysis (12-14). Flowchart 1 shows the screening process (Fig. 1). Three (4,13,14) of the four selected studies reported having been registered prior to patient recruitment.

- Characteristics of studies

The characteristics of the four included studies are shown in Table 2. The studies were conducted in four different countries. The randomized trials included 326 patients, and 294 patients completed the trials with a drop-out rate of 9.8% of the total number of patients. The number of patients varied from 40 to 117. In the four included studies, a single-implant surgery was performed. Regarding the area of implant placement, only one trial has reported its location that was the posterior region of the mandible (12).

The preemptive medication used were: 4mg dexamethasone (4), 600mg ibuprofen (4,14), 25mg dexketoprofen trometamol (13), and 40mg piroxicam (12), with placebo as control in all studies. In all trials, the participants were allowed to have analgesic rescue medication in any time they needed. There was no restriction regarding minimum time for taking the rescue medication neither for pain score, in a way that patients were free to decide whether to use it or not [4,154]. In two studies (12,13), analgesic and anti-inflammatory medication were prescribed to be taken regardless of the level of pain.

**Table 2:**
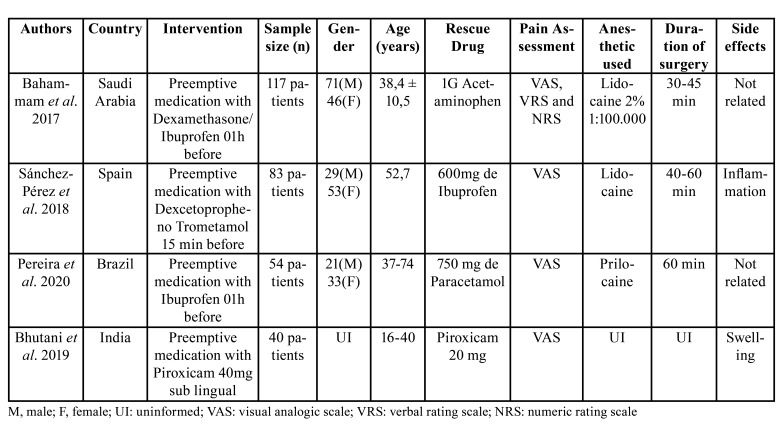
Characteristics of the included studies.

- Assessment of preemptive medication

The time for administering preemptive medication in most of the included studies was 1 hour before the procedure (4,12,14). In one study (13), preemptive medication was administered 15 min before surgical intervention. All studies used the Visual Analogic Scale (VAS) for pain assessment, one study also used the Numeric Rate Scale (NRS) (4) and one trial also used the Verbal Rating Scale (VRS) (4), thus, the comparative analysis of the level of pain between the studies, took into account the scores obtained by the VAS scale, common to all studies. Pain assessment times varied from the immediate postoperative period (13) to 07 days after the surgical procedure (4). The follow-up time of the included studies varied from 1 to 168 hours (mean time 78.4 hours).

- Side effects 

All studies collected data on side effects (12-14). The most common reported side effects were edema (12) that presented a significant decrease in the test group but not in the control group. In another study (13), significant differences between the test group and the control group for inflammation and bleeding were noted. The control group had a lower degree of inflammation; however, it obtained a higher degree of bleeding than the test group. No side effects like infections, major edema and bleeding were observed (14).

- Bias Risk Assessment

We contacted authors for risk of bias and data extraction when the information was unclear in the text, with only two (13,14) of the four authors responding. Table 3 presents the results for the assessment of risk of bias. All the studies that were analyzed showed "some concerns” on the final judgment.

- Meta-analysis of postoperative pain

**Figure 2:**
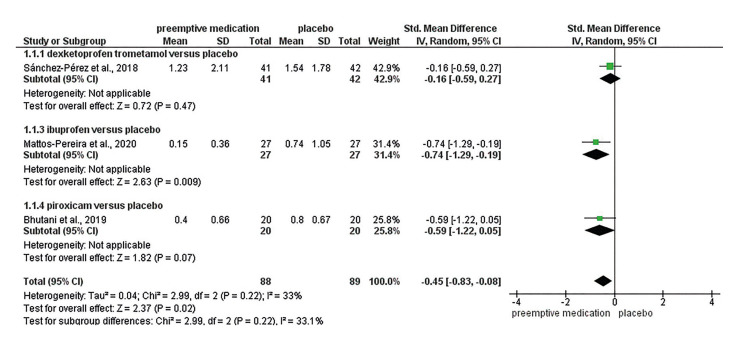
Forest plot for primary outcome 1-2 hours.

Fig. 2 shows the comparison between preemptive medication versus placebo 1-2 hours after the surgical procedure, divided in subgroups according to the type of drug.

**Table 3:**
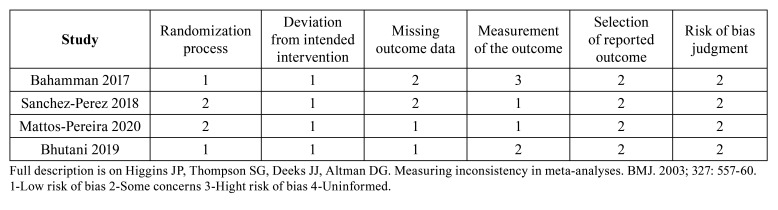
Risk of bias.

Overall pooled SMD showed that preemptive medication had a small effect in reducing pain (SMD: -0.45; IC: -0.83; -0.08) when compared to placebo, with low certainty of the evidence. However, only Ibuprofen had large effect compared to placebo (SMD: -0.74; CI: -1.29; -0.19).

**Figure 3:**
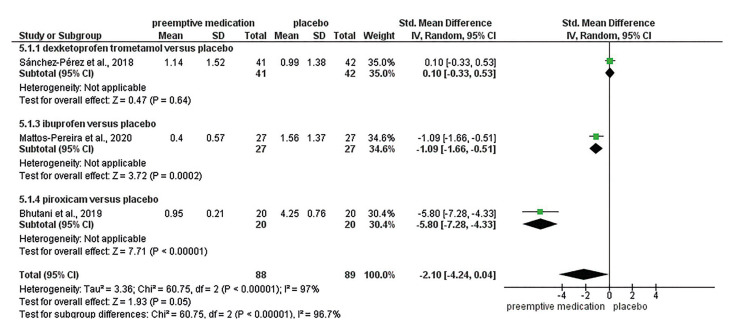
Forest plot for primary outcome 6-8 hours.

Fig. 3 shows the comparison between preemptive medication versus placebo 6-8 hours after the surgical procedure. Preemptive medication had a large effect (SMD = -2.10; 95%CI: -4.24; 0.04) when compared to placebo, with very low certainty of the evidence. Subgroup analysis showed that both ibuprofen (SMD = -1.09; 95%CI: -1.66; -0.51) and piroxicam (MD = -5.80; 95%CI: -7.28; -4.33) had a large effect when compared to placebo.

- Certainty of the evidence

**Figure 4:**
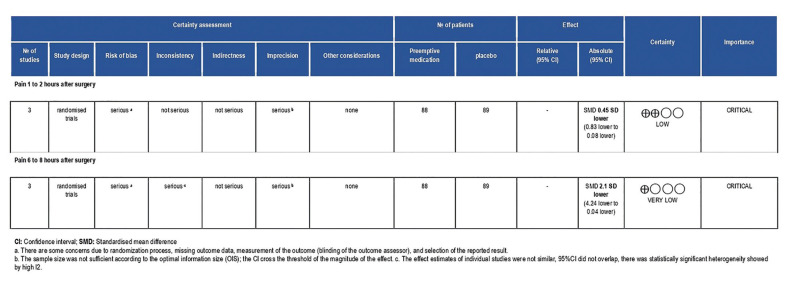
Summary of Finding (SoF) table showing the effect estimate together with the certainty of the evidence for pain 1-2 or 6-8 hours after the surgery.

The certainty of the evidence was rated down due to problems of risk of bias and imprecision for pain 1-2 hours after surgery. For 6-8 hours after surgery, the certainty of the evidence was rated down due to serious problems of risk of bias, inconsistency, and imprecision (Fig. 4).

## Discussion

The control of postoperative pain, edema and trismus has been the subject of continuous research in the area of oral and maxillofacial surgery, since pain can significantly reduce the patient's quality of life (3,15).

Tissue damage caused by the insertion of dental implants promotes the onset of an inflammatory process and, consequently, the release of several mediators such as prostaglandins (PGE) and bradykinins that sensitizes nociceptors (16). Anti-inflammatory drugs and local anesthetics inhibit the synthesis of mediators by inactivating the cyclooxygenase (COX) and phospholipase enzymes, altering nociception, and reducing peripheral sensitization. The decrease in the levels of inflammatory mediators at the site of tissue injury is strongly associated with these effects (17-19).

Our results showed that preemptive analgesia can slightly reduce pain 1-2 hours after implant surgery when compared to placebo. When assessing pain 6-8 hours after surgery, preventive analgesia proved to be effective, but the evidence was determined to be uncertain. Preemptive analgesia has been shown to be effective in reducing postoperative pain in the surgical removal of third molars (3,20), despite some studies (21,22) did not demonstrate a significant preemptive effect. Methodological differences may have an impact on these results, with divergence in the perception of postoperative pain. In dental implant placement, the preemptive analgesia protocol has considerably reduced the average pain scores, especially in the first 6 hours after the surgical procedure (3). A peak in prostaglandin E2 (PGE2) levels was observed approximately 3 hours after periodontal surgery and surgical removal of third molars (23,24). The peak of maximum pain at sites of acute inflammation usually occurs around 3-4 hours, coinciding with the maximum concentration of prostaglandins (22).

Our meta-analysis showed that the magnitude of the effect was bigger for 6-8 hours after surgery (large effect) compared to 1-2 hours after surgery (small effect). Among the drugs used in the evaluated RCTs, two studies (4,13) used Ibuprofen 600mg as preemptive medication. This non-steroidal anti-inflammatory drug (NSAID) inhibits the action of the COX enzyme, blocking the production of inflammatory mediators. It has a plasma half-life of 1 to 3 hours (25). The minimum plasma concentration of 26µg/ml of ibuprofen is capable of producing analgesic effect, and a dose of Ibuprofen of 600 mg has a maximum plasma level close to 69 µg / ml, on average, between 2 and 3 hours after the initial dose (26). Our subgroup analysis showed that ibuprofen had large effect 1-2 hours after surgery, when compared to placebo. Significant differences in pain level were observed in the first 4 hours between the ibuprofen group compared to the placebo group, emphasizing the beneficial effect of preemptive analgesia with ibuprofen (4). Unfavorable results were obtained with 25 mg of dexketoprofen trometamol (13), administered 15 min before the procedure for the period of 1-2 hours and 6-8 hours.

In a recent overview (27), it was shown that Ibuprofen 600mg had the highest proportion of patients (77%) who achieved at least 50% of maximum pain relief for 4-6 hours, followed by combinations of 400 mg of ibuprofen with 1,000 mg of acetaminophen (72%), 200 mg ibuprofen with 500 mg acetaminophen (69%). The evaluation of the duration of pain relief showed that 1000 mg of diflunisal, 650 mg of acetaminophen, 500 mg of diflunisal and 500 to 550 mg of naproxen, had the longest duration of action. The doses of 600 to 650 mg of acetaminophen, 25 mg of potassium diclofenac, 60 mg of codeine and 250 mg of gabapentin, were those that obtained the shortest duration of the analgesic effect.

The 25 mg dexketoprofen trometamol also obtained positive results for postoperative pain relief in third molar surgeries (28). In our meta-analysis, piroxican was effective 6-8 hours postoperative, when compared to placebo.

There was inconsistency for pain control after 6-8 hours due to the high I2, lack of overlap of the CIs and different individual effect estimates among studies (29). The heterogeneity may be explained by the different drugs used in the final pooled effect estimate. Moreover, there was serious imprecision for pain control after 1-2 hours and after 6-8 hours. In fact, the amplitude of CI showed that, for 1-2 hours, preemptive analgesia can have a moderate (lower CI: -0.71) to a trivial effect (upper CI: -0.15) compared to placebo. For 6-8 hours, preemptive analgesia can have a large (lower CI: -3.14) to a trivial effect (upper CI: -0.14) compared to placebo. Although in both cases the preemptive medication has a beneficial effect compared to placebo, the magnitude of the effect in reducing pain is too imprecise. In fact, pain is very subjective and can vary from patient to patient.

Preemptive analgesia can have more benefits than harm compared to placebo, as pain after oral and maxillofacial surgery can affect the patient’s quality of life (3,15). However, for implant surgery, the evidence is uncertain due to the variation of the magnitude of the effect. There were also serious problems due to the risk of bias. The clinical trials had some concerns due to randomization process (13,14), missing outcome data (4,13), and selection of reported outcome, as no study reported a registered protocol previously to conducting the trials (4,12-14). In addition, one studie had some concerns regarding blinding of the outcome assessor (12), while other study was at high risk of bias (4).

- Strengths and limitations:

This meta-analysis is limited due to the small number of included trials, which inputted imprecision on data. Furthermore, there was a variety of treatment protocols and methods for evaluating the results. One of the four selected studies could not be included in the meta-analysis as its data was provided in median values with non-normal distribution and not in mean values.

The present study has strengths as it uses the Cohen’s classification for the magnitude of the effect together with GRADE guidelines to interpret data for the clinical practice (11). The small important effect of preemptive analgesia within 1-2 hours leads us to question some protocols, such as the standardization of the anesthetic drug, the number of implants and the area where they will be placed, since the surgical trauma can influence the results of preemptive analgesia (3,15,27). The 4 studies included in this systematic review, (4,12,13,14) included only cases of single-implant surgery and only one study (12) reported the region where the implants were placed. It is important to note that this can impact the bias risk assessment of studies without this information. Two meta-analyzes (30,31) showed a better anesthetic effect of articaine 4% with epinephrine 1:100,000 compared to lidocaine 2% with epinephrine 1: 100,000. In addition, the anesthetic technique used can also influence the perception of postoperative pain and mask the effects of preemptive analgesia. The alveolar nerve block, for example, presents a greater residual postoperative analgesia, when compared to the infiltrative techniques (32). Studies evaluating preemptive analgesia should consider this data during methodological planning and results interpretation and discussion. Ideally, the test and control groups should be comparable in relation to the surgical sites, consequently, in relation to the anesthetic techniques used. These findings lead us to question whether the use of a more potent anesthetic base can mask the effects of preemptive medication. For pain after 6-8 hours, we observed that preemptive medication may have little effect on the expected result, but the evidence is very uncertain.

The study with the largest sample (13) did not demonstrate significant effects of preemptive analgesia in the evaluated postoperative periods. In the qualitative analysis, it demonstrated limitations in relation to bias due to deviation from intended intervention and missing outcome data. Meta-analyzes allow gathering the results of several studies with a common objective and, therefore, their results have greater power than results from individual studies.

Preemptive analgesia remains a very controversial, but a timely topic. There are several preventive analgesia protocols in surgical procedures for dental implant placement, with great methodological differences between clinical trials. Among these main differences, the following ones stand out: periods of postoperative pain assessment, methods of drug administration and differences in local anesthetics and vasoconstrictors.

The preemptive analgesia is effective for pain control. However, the magnitude of the effect is imprecise and can vary from large to trivial effect. For the clinical practice, there are two factors that can influence pain control: the time of drug administration and the time length of the surgery. When the drug is administered closer to the surgery, the drug effect can be extended for longer periods. Also, shorter surgeries can influence the perceived pain by the patient when compared to longer surgeries with longer intra-oral manipulation and more implants placement.

For future studies, RCTs should properly randomize patients and provide the blinding of the outcome assessor (the patient) with standardized pills for the intervention and control groups. Moreover, for more transparency in the research protocol, authors should register the trial before starting the study.

## Conclusions

The preemptive analgesia may slightly reduce pain from one to two hours after surgery when compared to patients who did not use preemptive medication. After six to eight hours, the preemptive analgesia may have a large effect in reducing pain compared to not using preemptive medication. However, the evidence is very uncertain and very imprecise.
